# The Impact of Anticholinergic Use for Overactive Bladder on Cognitive Changes in Adults with Normal Cognition, Mild Cognitive Impairment, or Dementia

**DOI:** 10.1016/j.euros.2022.10.008

**Published:** 2022-10-25

**Authors:** Blayne Welk, J. Andrew McClure

**Affiliations:** a Department of Surgery and Epidemiology and Biostatistics, Western University, London, Ontario, Canada; b London Health Sciences Centre, London, Ontario, Canada

**Keywords:** Anticholinergic medications, Dementia, Overactive bladder, Cognition

## Abstract

**Background:**

Few studies have addressed whether anticholinergic (AC) medications for overactive bladder (OAB) cause cognitive decline in individuals with existing cognitive impairment, and whether the *APOE* ε4 gene increases this risk.

**Objective:**

To determine whether OAB AC use is associated with a clinically relevant change in cognitive measures among adults with normal and abnormal cognition.

**Design, setting, and participants:**

This was a retrospective cohort study using data from the National Alzheimer’s Coordinating Center. Patients were enrolled at specialized centers in the USA between 2005 and 2019. Patients with existing OAB AC use, missing *APOE* ε4 status, and confounding neurologic diagnoses were excluded. New users of an OAB AC were matched 1:1 to patients not taking an OAB AC using propensity scores.

**Intervention:**

New use of oxybutynin, tolterodine, solifenacin, trospium, darifenacin, or fesoterodine.

**Outcome measurements and statistical analysis:**

The outcome was a change in cognitive function, measured as a ≥1-point increase on the Clinical Dementia Rating (CDR) instrument or a ≥3-point decrease on the Mini-Mental State Examination (MMSE). Conditional logistic regression with odds ratios (ORs) was conducted. We also tested for *APOE* ε4 effect modification.

**Results and limitations:**

Among 18 835 eligible patients, 782 matched pairs were identified. The most common OAB ACs were oxybutynin (38%) and tolterodine (23%). There was no significant increase in the risk of a clinically relevant cognitive decline among OAB AC users (CDR: OR 1.38, 95% confidence interval [CI] 0.93–2.05; *p* = 0.11, MMSE: OR 1.06, 95% CI 0.79–1.43; *p* = 0.70). There was no significant interaction between *APOE* ε4 status and OAB AC use for the CDR (*p* = 0.38) or MMSE (*p* = 0.95) outcomes. Users of oxybutynin or tolterodine had numerically higher odds of a change on the CDR test (OR 1.65, 95% CI 0.98–2.77) that was close to statistical significance (*p* = 0.06). Limitations include the inability to determine medication dose or duration, and residual confounding.

**Conclusions:**

OAB AC use was not associated with a significant change in cognitive function among individuals with normal and abnormal cognition. Further research is necessary to determine if oxybutynin and tolterodine are significantly more likely to cause cognitive decline.

**Patient summary:**

Use of a specific class of overactive bladder medication was not associated with negative changes in brain function among patients with either normal or abnormal function. A genetic risk factor for Alzheimer’s disease did not predispose individuals to cognitive decline when taking these drugs. Two of the drugs (oxybutynin and tolterodine) may lead to a higher risk of cognitive decline in comparison to other drugs, and this needs further research.

## Take Home Message

Use of anticholinergic medication for overactive bladder (OAB) was not associated with a significant decline in markers of cognitive function among a group of individuals with normal or impaired cognition. *APOE* ε4 allele status did not modify the effect of OAB anticholinergic medications on cognition.

## 1 Introduction

Overactive bladder (OAB) is a common condition, both in the general population and among those with cognitive dysfunction, and it increases in prevalence as people age [1]. OAB is defined as urinary urgency, usually with frequency and nocturia, and sometimes with urgency incontinence [2]. These symptoms are significant as they can lead to falls and fractures, skin breakdown, institutionalization, poor sleep, and impaired quality of life and work performance [1], [3], [4]. Initial treatment for OAB consists of behavioral changes and pharmacologic therapy [5]. Over the past 50 yr, several newer OAB anticholinergics (ACs) have been developed, and a new OAB medication class (β3 agonists) for the treatment of OAB symptoms has become commercially available over the past decade. However, a large proportion of patients continue to use some of the first ACs approved for OAB, such as oxybutynin, in part because of insurance reimbursement rules [6].

There is a large body of research suggesting that OAB AC medications (primarily oxybutynin and tolterodine) can cause cognitive dysfunction with short-term use; it has been shown that AC medications in general (and OAB ACs specifically) increase the risk of dementia [7], [8]. However, there is a need for more studies among individuals with pre-existing cognitive dysfunction [9]. In addition, the apolipoprotein (*APOE*) ε4 gene is a risk factor for Alzheimer’s disease [10] and may lead to greater sensitivity to AC-mediated cognitive decline [11]. To the best of our knowledge, *APOE* ε4 has not been evaluated as a possible risk factor for OAB AC-mediated cognitive decline. Our objective was to use data from the National Alzheimer’s Coordinating Center (NACC) to determine if OAB AC use is associated with an increase in the risk of cognitive decline, and to examine if *APOE* ε4 carrier status mediates any cognitive decline observed with OAB AC use.

## 2 Patients and methods

This is a retrospective, propensity score–matched, cohort study using prospective collected data from the NACC. The NACC started collecting their uniform data set via Alzheimer's Disease Research Centers (ADRCs) in the USA in 2005. Participants are enrolled using different techniques across these ADRCs. All participants provide written consent at the time of enrollment. Standardized data collection tools are used, and details of these have been described previously [12], [13]. As this was a secondary analysis of existing data, ethics review was not required.

We identified all participants who were enrolled in the NACC between September 1, 2005, and December 31, 2019. We excluded individuals if: they did not have at least one follow-up visit; their medication list was not available; they were already using an OAB AC medication on enrollment; they had a cognitive condition other than normal cognition, mild cognitive impairment, or dementia; they were missing genetic test results for *APOE* ε4 allele status; or there was >4 yr between their first and second visits. We excluded individuals with any unrelated or potentially confounding neurologic diagnoses: cognitive status categorized as “impaired not mild cognitive impairment”; frontotemporal dementia; hydrocephalus; progressive supranuclear palsy; primary progressive aphasia; corticobasal degeneration; prion disease; Huntington’s disease; Down’s syndrome; central nervous system cancer; traumatic brain injury; alcohol-related dementia; or dementia of undetermined etiology. Patients in the NACC were diagnosed with Alzheimer’s disease according to the criteria of the Alzheimer’s Association or the National Institute of Neurological and Communicative Disorders and Stroke/Alzheimer’s Disease and Related Disorders Association, and mild cognitive impairment was diagnosed according to on Petersen’s criteria [13].

Our primary exposure was defined as new use of an OAB AC medication (oxybutynin, tolterodine, solifenacin, trospium, darifenacin, or fesoterodine) at a follow-up NACC visit. Medication use was assessed at each visit by trained study staff who reviewed all the patient’s medication bottles during the study visit. We considered *APOE* ε3/ε4 or ε4/ε4 allele status as being positive for the *APOE* ε4 allele [10].

Our primary outcomes were changes on the Clinical Dementia Rating dementia staging instrument (CDR; scale from 0 = no dementia to 4 = severe cognitive impairment) [14] and the Mini-Mental state examination (MMSE; measures orientation, attention, memory, language, and visual-spatial skills, scored from 0 to 30; a lower score represents greater cognitive impairment) [15]. Secondary outcomes included the Boston naming test, the Wechsler Adult Intelligence Scale-revised (WAIS-R) digit substitution test, and the trail-making test part B [16]. These represent measures of memory and language, psychomotor speed, and executive function, respectively. A lower score on the Boston naming test or the WAIS-R digit substitution test, or a longer time to complete the trail-making test part B represent worse scores. In 2015, the NACC replaced the MMSE and the Boston naming test with the Montreal Cognitive Assessment (MOCA) and the multilingual naming test, respectively. Where necessary, we used the NACC Crosswalk Study [17] to convert scores between the MMSE and MOCA, and the Boston naming test and multilingual naming test.

### 2.1 Statistical analysis

We created a propensity score using 38 variables available in the NACC data set that may be relevant to cognition, including age, sex, language, years of education, marital status, vision or hearing impairment, and prevalent use of several medications (fully listed in the Supplementary material). Patients with new use of an OAB AC medication were matched 1:1 with participants without new use of an OAB AC medication according to the logit of the propensity score, NACC data era (before vs after 2015, owing to the new cognitive tests), total number of NACC visits, and cognitive status (normal, mild cognitive impairment, or dementia). Differences in baseline characteristics were measured using a *t* test or χ^2^ test, as appropriate.

Outcomes were compared between the visit before use of an OAB AC and the next follow-up visit at which new use of an OAB AC was identified. Changes in primary and secondary outcomes between visits were compared between the two groups using independent *t* tests. A conditional logistic regression model was used to determine if OAB AC medication exposure predicted a clinically important cognitive decline for our primary endpoints (≥1-point increase in CDR score, or a ≥3-point decrease in MMSE score, each tested as binary variable) [18]. An interaction term for APOE ε4 status (used as a binary variable) was tested in this model for significance. Two secondary analyses were explored. First, the primary outcome models were stratified by cognitive status (normal, mild cognitive impairment, or dementia). Second, the primary outcomes were stratified by OAB AC type (among matched pairs who were using oxybutynin or tolterodine, and among matched pairs who were using other OAB AC medications). Results from the conditional logistic regression models are reported as an odds ratio (OR) and 95% confidence interval (CI). The mean and standard deviation (SD) are reported. Pairs with missing data were excluded from the analyses as necessary. Statistical analysis was carried out using SAS Enterprise Guide v8.3 (SAS Institute, Cary, NC, USA). Two-sided *p* values <0.05 were considered significant.

## 3 Results

**Fig. 1:**
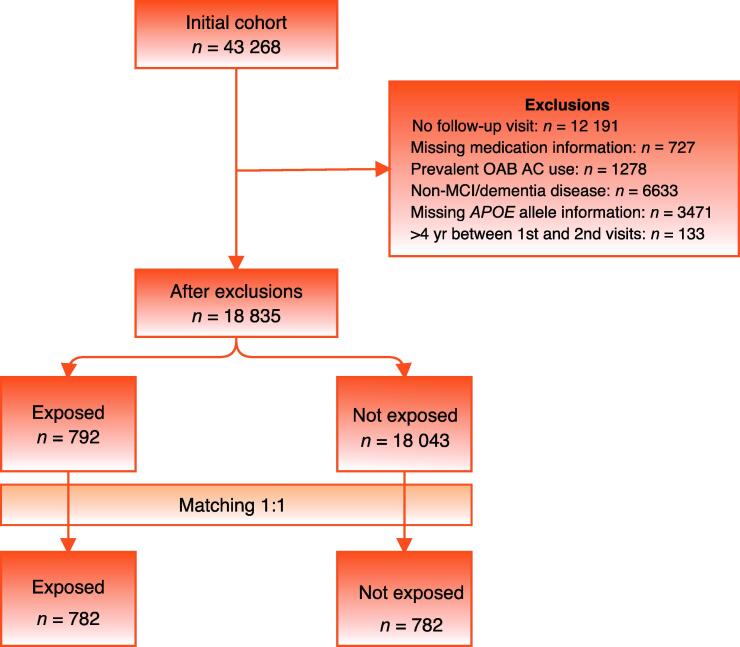
Flowchart showing the creation of the matched cohorts. AC = anticholinergic; MCI = mild cognitive impairment; OAB = overactive bladder.

Our initial cohort of 43 268 individuals was reduced to 18 835 after exclusions (Fig. 1). Before matching, some differences were noted between the OAB AC and non-OAB AC groups; the OAB AC group tended to be older, have more NACC visits, weigh more, use antihypertensives, use an anti-Parkinson medication, and have more hearing difficulty. We were able to successfully match 782 of the possible 792 participants who had newly started an OAB AC to 782 participants who did not start an OAB AC. After matching, all baseline characteristics measured were similar between the groups. Selected characteristics are listed in Table 1, with the full list of baseline characteristics before and after matching shown in the Supplementary material. The OAB AC medications used were oxybutynin (299/782, 38%), tolterodine (178/782, 23%), solifenacin (163/782, 21%), trospium (78/782, 10%), darifenacin (48/782, 6%), and fesoterodine (20/782, 3%).

**Table 1:** Selected baseline characteristics of the matched OAB AC users and nonusers

Variable	Non-OAB AC users	OAB AC users	*p* value
	(*n* = 782)	(*n* = 782)	
**General characteristics**			
Mean age at initial visit, yr (SD)	74.1 (9.0)	74.7 (8.4)	0.196
Male, *n* (%)	302 (38.6)	303 (38.7)	0.959
Mean time between baseline and FU visits, d (SD)	434 (138)	455 (165)	0.070
Cognitive status at study visit, *n* (%)			
Normal cognition	393 (50.3)	393 (50.3)	1.000
Mild cognitive impairment	173 (22.1)	173 (22.1)	
Dementia	216 (27.6)	216 (27.6)	
Education, *n* (%)			
<12 yr	45 (5.8)	51 (6.5)	0.496
12–13 yr	200 (25.6)	200 (25.6)	
14–16 yr	251 (32.1)	263 (33.6)	
≥17 yr	286 (36.6)	266 (34.0)	
Visual impairment, *n* (%)	234 (29.9)	256 (32.7)	0.484
Hearing impairment, *n* (%)	582 (74.4)	572 (73.1)	0.751
Living situation, *n* (%)			
Lives alone	202 (25.8)	209 (26.7)	0.445
Lives with spouse or partner	506 (64.7)	492 (62.9)	
Lives with relative or friend	60 (7.7)	56 (7.2)	
Other	14 (1.8)	25 (3.2)	
**Medication use, *n* (%)**			
Antihypertensives	433 (55.4)	444 (56.8)	0.575
Antiadrenergic agent	84 (10.7)	82 (10.5)	0.870
Calcium channel blocking agent	129 (16.5)	141 (18.0)	0.422
Diuretic	100 (12.8)	120 (15.3)	0.146
Lipid lowering medication	356 (45.5)	358 (45.8)	0.919
Anticoagulant or antiplatelet agent	272 (34.8)	277 (35.4)	0.791
Antidepressant	224 (28.6)	233 (29.8)	0.617
Antipsychotic agent	16 (2.0)	21 (2.7)	0.405
Anxiolytic, sedative, or hypnotic agent	98 (12.5)	105 (13.4)	0.598
Alzheimer’s disease medications	206 (26.3)	202 (25.8)	0.818
Anti-Parkinson agent	58 (7.4)	53 (6.8)	0.622
Estrogen hormone therapy	38 (4.9)	35 (4.5)	0.719
Diabetes medication	83 (10.6)	82 (10.5)	0.934
***APOE* genotype, *n* (%)**			
ε3/ε3	396 (50.6)	390 (49.9)	0.995
ε3/ε4	242 (30.9)	245 (31.3)	
ε3/ε2	73 (9.3)	77 (9.8)	
ε4/ε4	49 (6.3)	47 (6.0)	
ε4/ε2	20 (2.6)	20 (2.6)	
ε2/ε2	2 (0.3)	3 (0.4)	

AC = anticholinergic; FU = follow-up; OAB = overactive bladder.

**Fig. 2:**
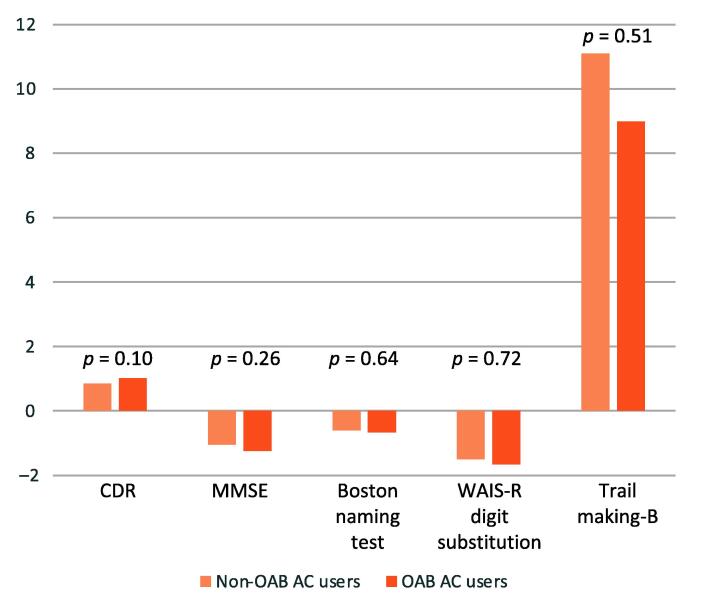
Comparison of the change in primary and secondary outcomes between the matched OAB AC and non-OAB AC users. The *p* values are from an independent *t* test for the change in score between the two groups. AC = anticholinergic; CDR = Clinical Dementia Rating; MMSE = Mini-Mental State Examination; OAB = overactive bladder; WAIS-R = Wechsler Adult Intelligence Scale-revised.

The mean time between the primary visit (when no OAB AC was being used) and the next follow-up visit (at which OAB AC use was newly identified) was 445 d (SD 153) and did not significantly differ between the groups. There was no significant difference in the change in primary or secondary outcomes between the groups (Table 2, Fig. 2). The proportion of participants who had a clinically significant change in CDR score (8.1% vs 10.1%) or MMSE score (20.7% vs 21.9%) was similar between the groups. When this was analyzed using a conditional logistic regression model, there was no significant increase in the risk of important cognitive decline for OAB AC use (CDR increase ≥1 point: OR 1.38, 95% CI 0.93–2.05; *p* = 0.11; MMSE decrease ≥3 points: OR 1.06, 95% CI 0.79–1.43; *p* = 0.70). There was no significant interaction between APOE ε4 carrier status and a clinically important cognitive decline measured with either the CDR (*p* = 0.38) or MMSE (*p* = 0.95). ORs were not statistically significant in any of the three individual cognitive groups (normal, mild cognitive impairment, dementia) for a clinically significant change in CDR or MMSE score (Table 3). There was an higher risk of a clinically important change in the CDR among users of oxybutynin or tolterodine (OR 1.65, 95% CI 0.98–2.77) that was close to statistical significance (*p* = 0.06); this increase in risk was not seen for users of the other ACs (OR 1.05, 95% CI 0.56–1.97; *p* = 0.87).

**Table 2:** Results for the primary and secondary outcomes

Test and group^a^	Mean score (standard deviation)		
	Baseline visit	Follow-up visit	Change
**Non-OAB AC users**			
MMSE	26.17 (4.71)	25.26 (5.70)	−1.05 (2.92)
CDR	2.37 (3.54)	3.21 (4.56)	+0.85 (1.97)
Boston name test	24.48 (6.06)	24.22 (6.53)	−0.60 (3.05)
WAIS-R digit substitution test	37.80 (16.01)	36.57 (17.18)	−1.50 (6.61)
Trail-making test part B	133.50 (82.23)	133.18 (85.78)	11.10 (52.12)
**OAB AC users**			
MMSE	26.22 (4.57)	25.10 (5.93)	−1.24 (3.21)
CDR	2.21 (3.25)	3.23 (4.43)	+1.02 (2.19)
Boston name test	24.83 (5.44)	24.34 (6.21)	−0.67 (2.73)
WAIS-R digit substitution test	36.75 (14.83)	35.76 (15.82)	−1.66 (6.97)
Trail-making test part B	139.73 (84.88)	142.53 (87.03)	+8.99 (53.50)

AC = anticholinergic; CDR = Clinical Dementia Rating; MMSE = Mini-Mental Sate Examination; OAB = overactive bladder; WAIS-R = Wechsler Adult Intelligence Scale-revised.

a Missing data for MMSE (207 individuals), Boston naming test (272 individuals), WAIS-R digit substitution test (694 individuals), and trail-making test part B (489 individuals).

**Table 3:** Primary outcomes from the conditional logistic regression of matched OAB anticholinergic users and nonusers (reference group) overall and stratified by cognitive status

Group	CDR		MMSE	
	OR (95% CI)	*p* value	OR (95% CI)	*p* value
Overall (*n* = 782 pairs)	1.38 (0.93–2.05)	0.11	1.06 (0.79–1.43)	0.70
Normal cognition (*n* = 393 pairs)	3.00 (0.61–14.86)	0.18	1.19 (0.61–2.31)	0.61
Mild cognitive impairment (*n* = 173 pairs)	0.64 (0.25–1.64)	0.35	0.90 (0.54–1.51)	0.69
Dementia (*n* = 216 pairs)	1.55 (0.97–2.48)	0.07	1.14 (0.73–1.77)	0.57

CDR = Clinical Dementia Rating; MMSE = Mini-Mental Sate Exam; CI = confidence interval; OAB = overactive bladder; OR = odds ratio.

## 4 Discussion

We did not find that new use of OAB AC medications was associated with a clinically important change in overall cognitive status. Similarly, three secondary outcome measures that tested memory and language, psychomotor speed, and executive function did not significantly different between OAB AC users and nonusers. Prior studies have shown that oxybutynin and tolterodine can have significant negative short-term cognitive effects [8], [9]; consistent with this, we found that when we stratified by AC type, users of oxybutynin and tolterodine had higher odds of a clinically significant CDR change compared to users of newer OAB ACs (solifenacin, trospium, fesoterodine, or darifenacin). This reinforces the need to examine continued use of these older OAB ACs now that newer agents are available. This continues to be relevant, as a study using Medicare data from 2013–2015 found that among individuals with dementia, 56% of new OAB AC users received oxybutynin as their incident OAB medication [19].

There are limited data on the cognitive impact of OAB AC use in individuals with dementia or cognitive impairment. A recent before/after observational study of 168 geriatric patients (of whom 12% had dementia) did not demonstrate a significant decline in MMSE scores on initiation of OAB AC medications (most commonly darifenacin and oxybutynin); however, there were significant improvements in depression, quality of life, and activities of daily living because of improved OAB symptom management [20]. A 28-d randomized, placebo-controlled study conducted in women with cognitive impairment (mean MMSE score of 15) found that oxybutynin (5 mg extended release once a day) did not impact cognitive measures, although the dose of oxybutynin may have been too low to have an effect on cognition [21]. Similarly, a three-arm crossover randomized placebo-controlled study showed that among older individuals with mild cognitive impairment (according to the Stockholm criteria), neither oxybutynin (10 mg immediate release) nor solifenacin (5 mg) impaired five standard measures of cognitive function; however, on secondary analysis, there was a significant decrease in the measures of power and continuity of attention 1–2 h after the oxybutynin dose [22]. Finally, in patients with dementia taking cholinesterase inhibitors, new use of oxybutynin or tolterodine resulted in a higher rate of functional decline than for patients with dementia who used cholinesterase inhibitors alone [23]. Taken together with the results from our study, evidence suggests that the use of oxybutynin in patients with dementia or mild cognitive impairment appears to have, at minimum, questionable cognitive safety.

In our study, *APOE* ε4 allele carrier status did not have a significant interaction with OAB AC use and a clinically significant cognitive decline. This is relevant, as approximately one-quarter of the population carry one of the *APOE* ε4 alleles. *APOE* ε4/ε4 homozygotes have an almost fivefold higher risk of dementia in comparison to noncarriers, and the cognitive effects of OAB AC use in individuals with this gene have not been clarified [24]. Mechanisms for the higher sensitivity to AC medications among *APOE* ε4 carriers include an altered blood-brain barrier that is more permeable to these medications, and lower levels of acetyltransferase enzymes [25]. Previous studies looking at APOE ε4 status and AC medications in general have reported mixed results. A registry of individuals with a family history of dementia found that overall AC burden significantly interacted with APOE ε4 status only for the change in score for the delayed recall portion of the auditory verbal learning test (one of seven cognitive outcomes in the study) [25]. A secondary analysis of a randomized controlled trial of nilvadipine among individuals with dementia found a significant interaction between *APOE* ε4 status and AC burden score on the CDR (one of three cognitive outcomes in the study) [26]. A cross-sectional study of adults aged >50 yr did not find that the cognitive effects of AC medication were different among carriers and noncarriers of APOE ε4 alleles [27].

Strengths of our study include the use of valid and sensitive measures of cognitive function, the use of a change threshold to define a clinically important cognitive decline (rather than simply a statistically significant numerical difference, which may be lower than the minimum clinically important difference of the instrument), and the creation of matched groups using a propensity score. Limitations of our research include the lack of data on the dose and duration of use for the OAB AC medications. This is because NACC medication data were collected as current medication utilization at discrete time points that were more than 1 yr apart. This means that some individuals may have used OAB AC medications between visits and then discontinued them before follow-up (and are therefore counted as nonusers in our study design, biasing our results toward the null hypothesis). OAB AC side effects, such as cognitive decline, or a lack of efficacy may have led to rapid discontinuation. In addition, some patients may have used these medications for only a short period of time, which is common for OAB medications [7], [28]. However, even in the setting of short-term use, side effects such as delirium and worsening cognitive function have been described [4], [29]. The next step would be to look at this research question using a different data set that includes both detailed medication utilization data (duration, dose) and relevant clinical measures of cognitive function. The sample sizes for many of the individual ACs were small, so we could not look at individual OAB medications. For example, the post hoc power of our evaluation of the risk of CDR decline in the oxybutynin/tolterodine group is estimated at 40%. Observational studies are always susceptible to residual confounding, and among those with normal cognition, individuals may have started OAB ACs because of early symptoms of dementia that had not manifested sufficiently for a diagnosis of mild cognitive impairment or dementia (leading to reverse causation). We had hoped to examine mirabegron as a second control group; however, the number of patients newly starting mirabegron in the NACC data set was too small. The NACC data set is not a random sample (eg, those with normal cognitive function tend to be more highly educated) and this should be considered when interpreting the study results and generalizing them to other populations. Finally, our secondary outcomes had a moderate level of missing data, which limited our statistical power.

## 5 Conclusions

This study provides a level of reassurance that the risk of clinically significant cognitive decline in a population with baseline cognitive dysfunction is not significantly associated with new use of OAB ACs. We did not find that *APOE* ε4 carrier status interacted with OAB AC use to modify their effect on cognitive measures. The odds of a clinically significant change in CDR score was higher for users of oxybutynin and tolterodine than for users of newer OAB AC medications (solifenacin, trospium, darifenacin, or fesoterodine); however as the statistical significance of this result was borderline, further research is necessary.

  ***Author contributions***: Blayne Welk had full access to all the data in the study and takes responsibility for the integrity of the data and the accuracy of the data analysis.

*Study concept and design*: McClure, Welk.

*Acquisition of data*: Welk.

*Analysis and interpretation of data*: McClure, Welk.

*Drafting of the manuscript*: Welk.

*Critical revision of the manuscript for important intellectual content*: McClure.

*Statistical analysis*: McClure.

*Obtaining funding*: None.

*Administrative, technical, or material support*: Welk.

*Supervision*: Welk.

*Other*: None.

  ***Financial disclosures:*** Blayne Welk certifies that all conflicts of interest, including specific financial interests and relationships and affiliations relevant to the subject matter or materials discussed in the manuscript (eg, employment/affiliation, grants or funding, consultancies, honoraria, stock ownership or options, expert testimony, royalties, or patents filed, received, or pending), are the following: Blayne Welk is a consultant for Beckton Dickinson outside the scope of this study. J. Andrew McClure has nothing to disclose.

  ***Funding/Support and role of the sponsor:*** None.

  ***Data sharing statement:*** The National Alzheimer’s Coordinating Center (NACC) data are available on request at www.naccdata.org. Details of the statistical analysis relevant to this paper are available from the corresponding author on reasonable request.

  ***Ethics considerations:*** This secondary analysis of existing data was exempt from ethics review.

  ***Acknowledgments:*** The NACC database is funded by National Institute on Aging (NIA)/National Institutes of Health grant U24 AG072122. NACC data are contributed by the following NIA-funded Alzheimer's Disease Research Centers: P30 AG062429 (PI James Brewer), P30 AG066468 (PI Oscar Lopez), P30 AG062421 (PI Bradley Hyman), P30 AG066509 (PI Thomas Grabowski), P30 AG066514 (PI Mary Sano), P30 AG066530 (PI Helena Chui), P30 AG066507 (PI Marilyn Albert), P30 AG066444 (PI John Morris), P30 AG066518 (PI Jeffrey Kaye), P30 AG066512 (PI Thomas Wisniewski), P30 AG066462 (PI Scott Small), P30 AG072979 (PI David Wolk), P30 AG072972 (PI Charles DeCarli), P30 AG072976 (PI Andrew Saykin), P30 AG072975 (PI David Bennett), P30 AG072978 (PI Neil Kowall), P30 AG072977 (PI Robert Vassar), P30 AG066519 (PI Frank LaFerla), P30 AG062677 (PI Ronald Petersen), P30 AG079280 (PI Eric Reiman), P30 AG062422 (PI Gil Rabinovici), P30 AG066511 (PI Allan Levey), P30 AG072946 (PI Linda Van Eldik), P30 AG062715 (PI Sanjay Asthana), P30 AG072973 (PI Russell Swerdlow), P30 AG066506 (PI Todd Golde), P30 AG066508 (PI Stephen Strittmatter), P30 AG066515 (PI Victor Henderson), P30 AG072947 (PI Suzanne Craft), P30 AG072931 (PI Henry Paulson), P30 AG066546 (PI Sudha Seshadri), P20 AG068024 (PI Erik Roberson), P20 AG068053 (PI Justin Miller), P20 AG068077 (PI Gary Rosenberg), P20 AG068082 (PI Angela Jefferson), P30 AG072958 (PI Heather Whitson, and P30 AG072959 (PI James Leverenz).

## References

[1] Stewart W.F., Rooyen J.B.V., Cundiff G.W. (2003). Prevalence and burden of overactive bladder in the United States. World J Urol.

[2] Haylen B.T., Ridder D.D., Freeman R.M. (2010). An International Urogynecological Association (IUGA)/International Continence Society (ICS) joint report on the terminology for female pelvic floor dysfunction. Int Urogynecol J.

[3] Coyne K.S., Sexton C.C., Thompson C.L. (2012). Impact of overactive bladder on work productivity. Urology.

[4] Welk B., Etaby K., McArthur E., Chou Q. (2022). The risk of delirium and falls or fractures with the use of overactive bladder anticholinergic medications. Neurourol Urodyn.

[5] Gormley E.A., Lightner D.J., Faraday M., Vasavada S.P. (2015). Diagnosis and treatment of overactive bladder (non-neurogenic) in adults: AUA/SUFU guideline amendment. J Urol.

[6] Chua K.J., Patel H.V., Tabakin A. (2021). Yearly trends of overactive bladder medication usage. Urology Pract.

[7] Welk B., McArthur E. (2020). Increased risk of dementia among patients with overactive bladder treated with an anticholinergic medication compared to a beta-3 agonist: a population-based cohort study. BJU Int.

[8] Duong V., Iwamoto A., Pennycuff J., Kudish B., Iglesia C. (2021). A systematic review of neurocognitive dysfunction with overactive bladder medications. Int Urogynecol J.

[9] Welk B., Richardson K., Panicker J.N. (2021). The cognitive effect of anticholinergics for patients with overactive bladder. Nat Rev Urol.

[10] Liu C.-C., Liu C.-C., Kanekiyo T., Xu H., Bu G. (2013). Apolipoprotein E and Alzheimer disease: risk, mechanisms and therapy. Nat Rev Neurol.

[11] Pomara N., Willoughby L.M., Wesnes K., Sidtis J.J. (2004). Increased anticholinergic challenge-induced memory impairment associated with the *APOE*-ε4 allele in the elderly: a controlled pilot study. Neuropsychopharmacology.

[12] Besser L., Kukull W., Knopman D.S. (2018). Version 3 of the National Alzheimer’s Coordinating Center’s Uniform Data Set. Alzheimer Dis Assoc Disord.

[13] Morris J.C., Weintraub S., Chui H.C. (2006). The Uniform Data Set (UDS): clinical and cognitive variables and descriptive data from Alzheimer disease centers. Alzheimer Dis Assoc Disord.

[14] Morris J.C. (1997). Clinical Dementia Rating: a reliable and valid diagnostic and staging measure for dementia of the Alzheimer type. Int Psychogeriatr.

[15] Mitchell A.J. (2009). A meta-analysis of the accuracy of the Mini-Mental State Examination in the detection of dementia and mild cognitive impairment. J Psychiatr Res.

[16] Faria C. de A., Alves H.V.D., Charchat-Fichman H. (2015). The most frequently used tests for assessing executive functions in aging. Dement Neuropsychol.

[17] Monsell S.E., Dodge H.H., Zhou X.-H. (2016). Results from the NACC Uniform Data Set Neuropsychological Battery Crosswalk Study. Alzheimer Dis Assoc Disord.

[18] Andrews J.S., Desai U., Kirson N.Y., Zichlin M.L., Ball D.E., Matthews B.R. (2019). Disease severity and minimal clinically important differences in clinical outcome assessments for Alzheimer’s disease clinical trials. Alzheimers Dement.

[19] Kachru N., Holmes H.M., Johnson M.L., Chen H., Aparasu R.R. (2021). Antimuscarinic use among older adults with dementia and overactive bladder: a Medicare beneficiaries study. Curr Med Res Opin.

[20] Esin E., Ergen A., Cankurtaran M. (2015). Influence of antimuscarinic therapy on cognitive functions and quality of life in geriatric patients treated for overactive bladder. Aging Ment Health.

[21] Lackner T.E., Wyman J.F., McCarthy T.C., Monigold M., Davey C. (2008). Randomized, placebo-controlled trial of the cognitive effect, safety, and tolerability of oral extended-release oxybutynin in cognitively impaired nursing home residents with urge urinary incontinence. J Am Geriatr Soc.

[22] Wagg A., Dale M., Tretter R., Stow B., Compion G. (2013). Randomised, multicentre, placebo-controlled, double-blind crossover study investigating the effect of solifenacin and oxybutynin in elderly people with mild cognitive impairment: the SENIOR study. Eur Urol.

[23] Sink K.M., Thomas J., Xu H., Craig B., Kritchevsky S., Sands L.P. (2008). Dual use of bladder anticholinergics and cholinesterase inhibitors: long-term functional and cognitive outcomes. J Am Geriatr Soc.

[24] Rasmussen K.L., Tybjærg-Hansen A., Nordestgaard B.G., Frikke-Schmidt R. (2018). Absolute 10-year risk of dementia by age, sex and APOE genotype: a population-based cohort study. Cand Med Assoc J.

[25] Collin B.G., Raju D., Katsikas S. (2021). The cognitive effects of anticholinergic drugs on apolipoprotein ε4 carriers and noncarriers in the Wisconsin Registry for Alzheimer’s Prevention study. Neuropsychology.

[26] Dyer A.H., Murphy C., Segurado R., Lawlor B., Kennelly S.P. (2019). NILVAD Study Group. Is ongoing anticholinergic burden associated with greater cognitive decline and dementia severity in mild to moderate Alzheimer’s disease?. J Gerontol A Biol Sci Med Sci.

[27] Limback-Stokin M.M., Krell-Roesch J., Roesler K. (2018). Anticholinergic medications and cognitive function in late midlife. Alzheimer Dis Assoc Disord.

[28] Song Y.S., Lee H.Y., Park J.J., Kim J.H. (2021). Persistence and adherence of anticholinergics and beta-3 agonist for the treatment of overactive bladder: systematic review and meta-analysis, and network meta-analysis. J Urol.

[29] Shiota T., Torimoto K., Momose H. (2014). Temporary cognitive impairment related to administration of newly developed anticholinergic medicines for overactive bladder: two case reports. BMC Res Notes.

