# Supplementary material for: The Impact of Anticholinergic Use for Overactive Bladder on Cognitive Changes in Adults with Normal Cognition, Mild Cognitive Impairment, or Dementia
Source: Eur Urol Open Sci. 2022 Oct 25;46:22–9. doi: 10.1016/j.euros.2022.10.008 (PMC9732452; doi:10.1016/j.euros.2022.10.008)
Supplement: Supplementary Data 1 [file mmc1.docx]

**Supplementary material**

**Supplementary Table 1 – Full unmatched baselines**

| **Variable** | **Value** | **Total** | | **Not Exposed** | | **Exposed** | | **p-value** |
| --- | --- | --- | --- | --- | --- | --- | --- | --- |
|  |  | **n = 18,835** | | **n = 18,043** | | **n = 792** | |  |
|  |  | Value | SD or Range | Value | SD or Range | Value | SD or Range |  |
| Age at initial visit | Mean (SD) | 71.8 | 10.1 | 71.7 | 10.1 | 74.6 | 8.3 | <.001 |
|  | Median (IQR) | 72 | 66 - 79 | 72 | 65 - 79 | 75 | 69 - 80 | <.0001 |
|  |  |  |  |  |  |  |  |  |
| Total number of study visits | Mean (SD) | 4.9 | 3.0 | 4.9 | 3.0 | 6.3 | 3.3 | <.001 |
|  | Median (IQR) | 4 | 3 - 6 | 4 | 3 - 6 | 6 | 4 - 8 | <.0001 |
|  |  |  |  |  |  |  |  |  |
| Height | Mean (SD) | 67.1 | 7.7 | 67.1 | 7.7 | 67.0 | 7.9 | 0.785 |
|  | Median (IQR) | 66 | 63 - 69.5 | 66 | 63 - 69.5 | 65.45 | 62.55 - 69 | 0.0207 |
|  |  |  |  |  |  |  |  |  |
| Weight | Mean (SD) | 200.9 | 160.4 | 200.0 | 158.9 | 220.3 | 189.8 | <.001 |
|  | Median (IQR) | 165 | 140 - 192 | 165 | 140 - 192 | 168 | 142 - 202 | 0.0023 |
|  |  |  |  |  |  |  |  |  |
| Cognitive status at study visit | Normal cognition | 9547 | 50.69% | 9154 | 50.73% | 393 | 49.62% | 0.276 |
|  | MCI | 3907 | 20.74% | 3725 | 20.65% | 182 | 22.98% |  |
|  | Dementia | 5381 | 28.57% | 5164 | 28.62% | 217 | 27.40% |  |
|  |  |  |  |  |  |  |  |  |
| Reason for coming to ADC | To participate in a research study | 14509 | 77.03% | 13896 | 77.02% | 613 | 77.40% | 0.002 |
|  | To have a clinical evaluation | 3390 | 18.00% | 3229 | 17.90% | 161 | 20.33% |  |
|  | Both research and evaluation or another reason | 919 | 4.88% | 901 | 4.99% | 18 | 2.27% |  |
|  | Unknown | 17 | 0.09% | 17 | 0.09% | 0 | 0.00% |  |
|  |  |  |  |  |  |  |  |  |
| Referral source | Non-professional contact | 6442 | 34.20% | 6180 | 34.25% | 262 | 33.08% | 0.138 |
|  | Professional contact | 6939 | 36.84% | 6663 | 36.93% | 276 | 34.85% |  |
|  | Other or Unknown | 5454 | 28.96% | 5200 | 28.82% | 254 | 32.07% |  |
|  |  |  |  |  |  |  |  |  |
| Sex | Men | 7817 | 41.50% | 7509 | 41.62% | 308 | 38.89% | 0.127 |
|  | Women | 11018 | 58.50% | 10534 | 58.38% | 484 | 61.11% |  |
|  |  |  |  |  |  |  |  |  |
| Primary language | English | 17617 | 93.53% | 16881 | 93.56% | 736 | 92.93% | 0.26 |
|  | Spanish | 801 | 4.25% | 758 | 4.20% | 43 | 5.43% |  |
|  | Mandarin | 93 | 0.49% | 93 | 0.52% | 0 | 0.00% |  |
|  | Cantonese | 52 | 0.28% | 50 | 0.28% | 2 | 0.25% |  |
|  | Russian | 7 | 0.04% | 7 | 0.04% | 0 | 0.00% |  |
|  | Japanese | 13 | 0.07% | 13 | 0.07% | 0 | 0.00% |  |
|  | Other or Unknown | 252 | 1.34% | 241 | 1.34% | 11 | 1.39% |  |
|  |  |  |  |  |  |  |  |  |
| Year of education | <12 | 1097 | 5.82% | 1046 | 5.80% | 51 | 6.44% | 0.023 |
|  | 12 to 13 | 4013 | 21.31% | 3810 | 21.12% | 203 | 25.63% |  |
|  | 14 to 16 | 6947 | 36.88% | 6681 | 37.03% | 266 | 33.59% |  |
|  | 17+ | 6698 | 35.56% | 6428 | 35.63% | 270 | 34.09% |  |
|  | Missing | 80 | 0.42% | 78 | 0.43% | 2 | 0.25% |  |
|  |  |  |  |  |  |  |  |  |
| Marital status | Married | 12073 | 64.10% | 11573 | 64.14% | 500 | 63.13% | 0.008 |
|  | Widowed | 3191 | 16.94% | 3021 | 16.74% | 170 | 21.46% |  |
|  | Divorced | 2057 | 10.92% | 1984 | 11.00% | 73 | 9.22% |  |
|  | Separated | 163 | 0.87% | 159 | 0.88% | 4 | 0.51% |  |
|  | Never married | 901 | 4.78% | 867 | 4.81% | 34 | 4.29% |  |
|  | Living as married/domestic partner | 325 | 1.73% | 317 | 1.76% | 8 | 1.01% |  |
|  | Other or unknown | 125 | 0.66% | 122 | 0.68% | 3 | 0.38% |  |
|  |  |  |  |  |  |  |  |  |
| Living situation | Lives alone | 4581 | 24.32% | 4370 | 24.22% | 211 | 26.64% | 0.353 |
|  | Lives with spouse or partner | 11982 | 63.62% | 11483 | 63.64% | 499 | 63.01% |  |
|  | Lives with relative or friend | 1488 | 7.90% | 1431 | 7.93% | 57 | 7.20% |  |
|  | Lives with group | 496 | 2.63% | 480 | 2.66% | 16 | 2.02% |  |
|  | Other or Unknown | 288 | 1.53% | 279 | 1.55% | 9 | 1.14% |  |
|  |  |  |  |  |  |  |  |  |
| Independence | Able to live independently | 13405 | 71.17% | 12852 | 71.23% | 553 | 69.82% | 0.061 |
|  | Requires some assistance with complex activities | 3823 | 20.30% | 3645 | 20.20% | 178 | 22.47% |  |
|  | Requires some assistance with basic activities | 1204 | 6.39% | 1151 | 6.38% | 53 | 6.69% |  |
|  | Completely dependent | 315 | 1.67% | 311 | 1.72% | 4 | 0.51% |  |
|  | Unknown | 88 | 0.47% | 84 | 0.47% | 4 | 0.51% |  |
|  |  |  |  |  |  |  |  |  |
| Type of residence | Single- or multi-family private residence | 17118 | 90.88% | 16427 | 91.04% | 691 | 87.25% | 0.001 |
|  | Retirement community or independent group living | 1076 | 5.71% | 1012 | 5.61% | 64 | 8.08% |  |
|  | Assisted living, adult family home, or boarding home | 255 | 1.35% | 235 | 1.30% | 20 | 2.53% |  |
|  | Skilled nursing facility, nursing home, hospital, or hospice | 110 | 0.58% | 106 | 0.59% | 4 | 0.51% |  |
|  | Other or unknown | 276 | 1.47% | 263 | 1.46% | 13 | 1.64% |  |
|  |  |  |  |  |  |  |  |  |
| Handedness | Left | 1509 | 8.01% | 1449 | 8.03% | 60 | 7.58% | 0.335 |
|  | Right | 16886 | 89.65% | 16175 | 89.65% | 711 | 89.77% |  |
|  | Ambidextrous | 369 | 1.96% | 354 | 1.96% | 15 | 1.89% |  |
|  | Unknown | 71 | 0.38% | 65 | 0.36% | 6 | 0.76% |  |
|  |  |  |  |  |  |  |  |  |
| Race | White | 15422 | 81.88% | 14755 | 81.78% | 667 | 84.22% | 0.132 |
|  | Black or African American | 2180 | 11.57% | 2093 | 11.60% | 87 | 10.98% |  |
|  | American Indian or Alaska Native | 70 | 0.37% | 67 | 0.37% | 3 | 0.38% |  |
|  | Native Hawaiian or Pacific Islander | 9 | 0.05% | 8 | 0.04% | 1 | 0.13% |  |
|  | Asian | 442 | 2.35% | 431 | 2.39% | 11 | 1.39% |  |
|  | Multiracial | 529 | 2.81% | 516 | 2.86% | 13 | 1.64% |  |
|  | Unknown or ambiguous | 183 | 0.97% | 173 | 0.96% | 10 | 1.26% |  |
|  |  |  |  |  |  |  |  |  |
| Antihypertensive or blood pressure medication | Yes | 9558 | 50.75% | 9108 | 50.48% | 450 | 56.82% | <.001 |
|  |  |  |  |  |  |  |  |  |
| Antihypertensive combination therapy | Yes | 881 | 4.68% | 827 | 4.58% | 54 | 6.82% | 0.004 |
|  |  |  |  |  |  |  |  |  |
| Angiotensin converting enzyme | Yes | 3007 | 15.96% | 2867 | 15.89% | 140 | 17.68% | 0.179 |
|  |  |  |  |  |  |  |  |  |
| Antiadrenergic agent | Yes | 1401 | 7.44% | 1316 | 7.29% | 85 | 10.73% | <.001 |
|  |  |  |  |  |  |  |  |  |
| Betaadrenergic blocking agent | Yes | 3530 | 18.74% | 3359 | 18.62% | 171 | 21.59% | 0.036 |
|  |  |  |  |  |  |  |  |  |
| Calcium channel blocking agent | Yes | 2536 | 13.46% | 2394 | 13.27% | 142 | 17.93% | <.001 |
|  |  |  |  |  |  |  |  |  |
| Diuretic | Yes | 2691 | 14.29% | 2571 | 14.25% | 120 | 15.15% | 0.478 |
|  |  |  |  |  |  |  |  |  |
| Vasodilator | Yes | 274 | 1.45% | 261 | 1.45% | 13 | 1.64% | 0.654 |
|  |  |  |  |  |  |  |  |  |
| Angiotensin II inhibitor | Yes | 1912 | 10.15% | 1829 | 10.14% | 83 | 10.48% | 0.754 |
|  |  |  |  |  |  |  |  |  |
| Lipid lowering medication | Yes | 7745 | 41.12% | 7383 | 40.92% | 362 | 45.71% | 0.007 |
|  |  |  |  |  |  |  |  |  |
| Nonsteroidal anti-inflammatory medication | Yes | 6854 | 36.39% | 6565 | 36.39% | 289 | 36.49% | 0.952 |
|  |  |  |  |  |  |  |  |  |
| Anticoagulant or antiplatelet agent | Yes | 6317 | 33.54% | 6036 | 33.45% | 281 | 35.48% | 0.237 |
|  |  |  |  |  |  |  |  |  |
| Antidepressant | Yes | 4929 | 26.17% | 4687 | 25.98% | 242 | 30.56% | 0.004 |
|  |  |  |  |  |  |  |  |  |
| Antipsychotic agent | Yes | 457 | 2.43% | 435 | 2.41% | 22 | 2.78% | 0.511 |
|  |  |  |  |  |  |  |  |  |
| Anxiolytic, sedative, or hypnotic agent | Yes | 2129 | 11.30% | 2020 | 11.20% | 109 | 13.76% | 0.026 |
|  |  |  |  |  |  |  |  |  |
| Medication for Alzheimer’s disease symptoms | Yes | 4835 | 25.67% | 4632 | 25.67% | 203 | 25.63% | 0.98 |
|  |  |  |  |  |  |  |  |  |
| Antiparkinson agent | Yes | 694 | 3.68% | 634 | 3.51% | 60 | 7.58% | <.001 |
|  |  |  |  |  |  |  |  |  |
| Estrogen hormone therapy | Yes | 720 | 3.82% | 684 | 3.79% | 36 | 4.55% | 0.278 |
|  |  |  |  |  |  |  |  |  |
| Estrogen + progestin hormone therapy | Yes | 111 | 0.59% | 108 | 0.60% | 3 | 0.38% | 0.429 |
|  |  |  |  |  |  |  |  |  |
| Diabetes medication | Yes | 1710 | 9.08% | 1628 | 9.02% | 82 | 10.35% | 0.202 |
|  |  |  |  |  |  |  |  |  |
| Vision | No | 12596 | 66.88% | 12077 | 66.93% | 519 | 65.53% | 0.573 |
|  | Yes | 5862 | 31.12% | 5603 | 31.05% | 259 | 32.70% |  |
|  | Unknown or Missing | 377 | 2.00% | 363 | 2.01% | 14 | 1.77% |  |
|  |  |  |  |  |  |  |  |  |
| Hearing | No | 3903 | 20.72% | 3704 | 20.53% | 199 | 25.13% | 0.008 |
|  | Yes | 14593 | 77.48% | 14014 | 77.67% | 579 | 73.11% |  |
|  | Unknown or Missing | 339 | 1.80% | 325 | 1.80% | 14 | 1.77% |  |
|  |  |  |  |  |  |  |  |  |
| APOE genotype | e3,e3 | 9176 | 48.72% | 8780 | 48.66% | 396 | 50.00% | 0.609 |
|  | e3,e4 | 6062 | 32.18% | 5814 | 32.22% | 248 | 31.31% |  |
|  | e3,e2 | 1660 | 8.81% | 1582 | 8.77% | 78 | 9.85% |  |
|  | e4,e4 | 1369 | 7.27% | 1322 | 7.33% | 47 | 5.93% |  |
|  | e4,e2 | 503 | 2.67% | 483 | 2.68% | 20 | 2.53% |  |
|  | e2,e2 | 65 | 0.35% | 62 | 0.34% | 3 | 0.38% |  |
|  |  |  |  |  |  |  |  |  |

**Supplementary Table 2 – Full matched baselines**

| **Variable** | **Value** | **Total** | **Not Exposed** | **Exposed** | **p-value** |
| --- | --- | --- | --- | --- | --- |
|  |  | **n = 1,564** | **n = 782** | **n = 782** |  |
|  |  |  |  |  |  |
| Age at initial visit | Mean (SD) | 74.4 ± 8.7 | 74.1 ± 9.0 | 74.7 ± 8.4 | 0.196 |
|  | Median (IQR) | 75 (68.5 - 81) | 74 (68 - 81) | 75 (69 - 80) | 0.145 |
|  |  |  |  |  |  |
| Total number of study visits* | Mean (SD) | 6.3 ± 3.3 | 6.3 ± 3.3 | 6.3 ± 3.3 | 1.000 |
|  | Median (IQR) | 6 (4 - 8) | 6 (4 - 8) | 6 (4 - 8) | 1.000 |
|  |  |  |  |  |  |
| Height | Mean (SD) | 66.9 ± 8.3 | 66.9 ± 8.6 | 67.0 ± 7.9 | 0.803 |
|  | Median (IQR) | 65.5 (62.5 - 69) | 65.5 (62.5 - 69) | 65.45 (62.5 - 69) | 0.922 |
|  |  |  |  |  |  |
| Weight | Mean (SD) | 217.2 ± 188.2 | 215.2 ± 188.7 | 219.1 ± 187.9 | 0.678 |
|  | Median (IQR) | 165 (140 - 198) | 164 (139 - 193) | 168 (142 - 202) | 0.068 |
|  |  |  |  |  |  |
| Cognitive status at study visit | Normal cognition | 786 (50.3%) | 393 (50.3%) | 393 (50.3%) | 1.000 |
|  | MCI | 346 (22.1%) | 173 (22.1%) | 173 (22.1%) |  |
|  | Dementia | 432 (27.6%) | 216 (27.6%) | 216 (27.6%) |  |
|  |  |  |  |  |  |
| Reason for coming to ADC | To participate in a research study | 1,234 (78.9%) | 627 (80.2%) | 607 (77.6%) | 0.305 |
|  | To have a clinical evaluation | 291 (18.6%) | 134 (17.1%) | 157 (20.1%) |  |
|  | Both research and evaluation or another reason | 39 (2.5%) | 21 (2.7%) | 18 (2.3%) |  |
|  |  |  |  |  |  |
| Referral source | Non-professional contact | 529 (33.8%) | 270 (34.5%) | 259 (33.1%) | 0.789 |
|  | Professional contact | 540 (34.5%) | 270 (34.5%) | 270 (34.5%) |  |
|  | Other or Unknown | 495 (31.6%) | 242 (30.9%) | 253 (32.4%) |  |
|  |  |  |  |  |  |
| Sex | Men | 605 (38.7%) | 302 (38.6%) | 303 (38.7%) | 0.959 |
|  | Women | 959 (61.3%) | 480 (61.4%) | 479 (61.3%) |  |
|  |  |  |  |  |  |
| Primary language | English | 1,449 (92.6%) | 721 (92.2%) | 728 (93.1%) | 0.854 |
|  | Spanish | 92 (5.9%) | 50 (6.4%) | 42 (5.4%) |  |
|  | Cantonese | 4 (0.3%) | 2 (0.3%) | 2 (0.3%) |  |
|  | Other or Unknown | 19 (1.2%) | 9 (1.2%) | 10 (1.3%) |  |
|  |  |  |  |  |  |
| Year of education | <12 | 96 (6.1%) | 45 (5.8%) | 51 (6.5%) | 0.496 |
|  | 12 to 13 | 400 (25.6%) | 200 (25.6%) | 200 (25.6%) |  |
|  | 14 to 16 | 514 (32.9%) | 251 (32.1%) | 263 (33.6%) |  |
|  | 17+ | 552 (35.3%) | 286 (36.6%) | 266 (34.0%) |  |
|  | Missing | 2 (0.1%) | 0 (0.0%) | 2 (0.3%) |  |
|  |  |  |  |  |  |
| Marital status | Married | 1,003 (64.1%) | 510 (65.2%) | 493 (63.0%) | 0.909 |
|  | Widowed | 331 (21.2%) | 163 (20.8%) | 168 (21.5%) |  |
|  | Divorced | 135 (8.6%) | 63 (8.1%) | 72 (9.2%) |  |
|  | Separated | 10 (0.6%) | 6 (0.8%) | 4 (0.5%) |  |
|  | Never married | 66 (4.2%) | 32 (4.1%) | 34 (4.3%) |  |
|  | Living as married/domestic partner | 13 (0.8%) | 5 (0.6%) | 8 (1.0%) |  |
|  | Other or unknown | 6 (0.4%) | 3 (0.4%) | 3 (0.4%) |  |
|  |  |  |  |  |  |
| Living situation | Lives alone | 411 (26.3%) | 202 (25.8%) | 209 (26.7%) | 0.445 |
|  | Lives with spouse or partner | 998 (63.8%) | 506 (64.7%) | 492 (62.9%) |  |
|  | Lives with relative or friend | 116 (7.4%) | 60 (7.7%) | 56 (7.2%) |  |
|  | Lives with group | 24 (1.5%) | 8 (1.0%) | 16 (2.0%) |  |
|  | Other or Unknown | 15 (1.0%) | 6 (0.8%) | 9 (1.2%) |  |
|  |  |  |  |  |  |
| Independence | Able to live independently | 1,104 (70.6%) | 556 (71.1%) | 548 (70.1%) | 0.679 |
|  | Requires some assistance with complex activities | 349 (22.3%) | 175 (22.4%) | 174 (22.3%) |  |
|  | Requires some assistance with basic activities | 101 (6.5%) | 48 (6.1%) | 53 (6.8%) |  |
|  | Completely dependent | 5 (0.3%) | 1 (0.1%) | 4 (0.5%) |  |
|  | Unknown | 5 (0.3%) | 2 (0.3%) | 3 (0.4%) |  |
|  |  |  |  |  |  |
| Type of residence | Single- or multi-family private residence | 1,387 (88.7%) | 704 (90.0%) | 683 (87.3%) | 0.538 |
|  | Retirement community or independent group living | 111 (7.1%) | 49 (6.3%) | 62 (7.9%) |  |
|  | Assisted living, adult family home, or boarding home | 36 (2.3%) | 16 (2.0%) | 20 (2.6%) |  |
|  | Skilled nursing facility, nursing home, hospital, or hospice | 6 (0.4%) | 2 (0.3%) | 4 (0.5%) |  |
|  | Other or unknown | 24 (1.5%) | 11 (1.4%) | 13 (1.7%) |  |
|  |  |  |  |  |  |
| Handedness | Left | 128 (8.2%) | 69 (8.8%) | 59 (7.5%) | 0.765 |
|  | Right | 1,399 (89.5%) | 696 (89.0%) | 703 (89.9%) |  |
|  | Ambidextrous | 27 (1.7%) | 12 (1.5%) | 15 (1.9%) |  |
|  | Unknown | 10 (0.6%) | 5 (0.6%) | 5 (0.6%) |  |
|  |  |  |  |  |  |
| Race | White | 1,325 (84.7%) | 665 (85.0%) | 660 (84.4%) | 0.998 |
|  | Black or African American | 169 (10.8%) | 82 (10.5%) | 87 (11.1%) |  |
|  | American Indian or Alaska Native | 6 (0.4%) | 3 (0.4%) | 3 (0.4%) |  |
|  | Native Hawaiian or Pacific Islander | 2 (0.1%) | 1 (0.1%) | 1 (0.1%) |  |
|  | Asian | 20 (1.3%) | 11 (1.4%) | 9 (1.2%) |  |
|  | Multiracial | 25 (1.6%) | 12 (1.5%) | 13 (1.7%) |  |
|  | Unknown or ambiguous | 17 (1.1%) | 8 (1.0%) | 9 (1.2%) |  |
|  |  |  |  |  |  |
| Antihypertensive or blood pressure medication | Yes | 877 (56.1%) | 433 (55.4%) | 444 (56.8%) | 0.575 |
|  |  |  |  |  |  |
| Antihypertensive combination therapy | Yes | 108 (6.9%) | 55 (7.0%) | 53 (6.8%) | 0.842 |
|  |  |  |  |  |  |
| Angiotensin converting enzyme | Yes | 268 (17.1%) | 129 (16.5%) | 139 (17.8%) | 0.502 |
|  |  |  |  |  |  |
| Antiadrenergic agent | Yes | 166 (10.6%) | 84 (10.7%) | 82 (10.5%) | 0.870 |
|  |  |  |  |  |  |
| Betaadrenergic blocking agent | Yes | 354 (22.6%) | 185 (23.7%) | 169 (21.6%) | 0.334 |
|  |  |  |  |  |  |
| Calcium channel blocking agent | Yes | 270 (17.3%) | 129 (16.5%) | 141 (18.0%) | 0.422 |
|  |  |  |  |  |  |
| Diuretic | Yes | 220 (14.1%) | 100 (12.8%) | 120 (15.3%) | 0.146 |
|  |  |  |  |  |  |
| Vasodilator | Yes | 23 (1.5%) | 11 (1.4%) | 12 (1.5%) | 0.834 |
|  |  |  |  |  |  |
| Angiotensin II inhibitor | Yes | 165 (10.5%) | 85 (10.9%) | 80 (10.2%) | 0.681 |
|  |  |  |  |  |  |
| Lipid lowering medication | Yes | 714 (45.7%) | 356 (45.5%) | 358 (45.8%) | 0.919 |
|  |  |  |  |  |  |
| Nonsteroidal anti-inflammatory medication | Yes | 570 (36.4%) | 286 (36.6%) | 284 (36.3%) | 0.916 |
|  |  |  |  |  |  |
| Anticoagulant or antiplatelet agent | Yes | 549 (35.1%) | 272 (34.8%) | 277 (35.4%) | 0.791 |
|  |  |  |  |  |  |
| Antidepressant | Yes | 457 (29.2%) | 224 (28.6%) | 233 (29.8%) | 0.617 |
|  |  |  |  |  |  |
| Antipsychotic agent | Yes | 37 (2.4%) | 16 (2.0%) | 21 (2.7%) | 0.405 |
|  |  |  |  |  |  |
| Anxiolytic, sedative, or hypnotic agent | Yes | 203 (13.0%) | 98 (12.5%) | 105 (13.4%) | 0.598 |
|  |  |  |  |  |  |
| Medication for Alzheimer’s disease symptoms | Yes | 408 (26.1%) | 206 (26.3%) | 202 (25.8%) | 0.818 |
|  |  |  |  |  |  |
| Antiparkinson agent | Yes | 111 (7.1%) | 58 (7.4%) | 53 (6.8%) | 0.622 |
|  |  |  |  |  |  |
| Estrogen hormone therapy | Yes | 73 (4.7%) | 38 (4.9%) | 35 (4.5%) | 0.719 |
|  |  |  |  |  |  |
| Estrogen + progestin hormone therapy | Yes | 6 (0.4%) | 3 (0.4%) | 3 (0.4%) | 1.000 |
|  |  |  |  |  |  |
| Diabetes medication | Yes | 165 (10.5%) | 83 (10.6%) | 82 (10.5%) | 0.934 |
|  |  |  |  |  |  |
| Vision | No | 1,046 (66.9%) | 534 (68.3%) | 512 (65.5%) | 0.484 |
|  | Yes | 490 (31.3%) | 234 (29.9%) | 256 (32.7%) |  |
|  | Unknown or Missing | 28 (1.8%) | 14 (1.8%) | 14 (1.8%) |  |
|  |  |  |  |  |  |
| Hearing | No | 385 (24.6%) | 189 (24.2%) | 196 (25.1%) | 0.751 |
|  | Yes | 1,154 (73.8%) | 582 (74.4%) | 572 (73.1%) |  |
|  | Unknown or Missing | 25 (1.6%) | 11 (1.4%) | 14 (1.8%) |  |
|  |  |  |  |  |  |
| APOE genotype | e3,e3 | 786 (50.3%) | 396 (50.6%) | 390 (49.9%) | 0.995 |
|  | e3,e4 | 487 (31.1%) | 242 (30.9%) | 245 (31.3%) |  |
|  | e3,e2 | 150 (9.6%) | 73 (9.3%) | 77 (9.8%) |  |
|  | e4,e4 | 96 (6.1%) | 49 (6.3%) | 47 (6.0%) |  |
|  | e4,e2 | 40 (2.6%) | 20 (2.6%) | 20 (2.6%) |  |
|  | e2,e2 | 5 (0.3%) | 2 (0.3%) | 3 (0.4%) |  |
|  |  |  |  |  |  |
| Accrual period | Pre-2015 | 1,404 (89.8%) | 702 (89.8%) | 702 (89.8%) | 1 |
|  | Post-2015 | 160 (10.2%) | 80 (10.2%) | 80 (10.2%) |  |
